# Costs of shoulder pain in primary care consulters: a prospective cohort study in The Netherlands

**DOI:** 10.1186/1471-2474-7-83

**Published:** 2006-11-01

**Authors:** Ton Kuijpers, Maurits W van Tulder, Geert JMG van der Heijden, Lex M Bouter, Daniëlle AWM van der Windt

**Affiliations:** 1 Institute for Research in Extramural Medicine, VU University Medical Center, Amsterdam, The Netherlands; 2 Department of Allied Health Care Research, Amsterdam School of Allied Health Care Education, The Netherlands; 3 Care and Public Health Research Institute, Maastricht University, The Netherlands; 4 Department of Health Economics and Health Technology Assessment, Institute of Health Sciences, Faculty of Earth and Life Sciences, VU University, Amsterdam, The Netherlands; 5 Julius Center for Health Sciences and Primary Care, University Medical Center, Utrecht, The Netherlands; 6 Primary Care Musculoskeletal Research Centre, Keele University, Keele Staffordshire, UK

## Abstract

**Background:**

Shoulder pain is common in primary care, and has an unfavourable outcome in many patients. Information on the costs associated with health care use and loss of productivity in patients with shoulder pain is very scarce. The objective of this study was to determine shoulder pain related costs during the 6 months after first consultation in general practice

**Methods:**

A prospective cohort study consisting of 587 patients with a new episode of shoulder pain was conducted with a follow-up period of 6 months. Data on costs were collected by means of a cost diary during 6 months.

**Results:**

84% of the patients completed all cost diaries. The mean consumption of direct health care and non-health related care was low. During 6 months after first consultation for shoulder pain, the mean total costs a patient generated were €689. Almost 50% of this total concerned indirect costs, caused by sick leave from paid work. A small proportion (12%) of the population generated 74% of the total costs.

**Conclusion:**

The total costs in the 6 months after first consultation for shoulder pain in primary care, mostly generated by a small part of the population, are not alarmingly high.

## Background

Shoulder pain is common with a one-year prevalence ranging between 5% and 47%[1-6]. The prevalence in the general population in The Netherlands has recently been estimated at 17% [7]. The annual incidence of shoulder pain in Dutch general practice ranges between 12 and 25/1000/year [7-9]. Shoulder pain has an unfavourable outcome in many patients. About 40 to 50% of all patients who present with a new episode of shoulder pain in primary care report persistent symptoms after 6 to 12 months [10-12].

Musculoskeletal disorders are the second most expensive disease group for health care costs in the Netherlands, and represent 6% of the total healthcare costs [13]. Information on the costs associated with health care use and loss of productivity in patients with shoulder pain is very scarce, especially for the large majority of patients who are treated in primary health care.

We performed a cohort study among patients who presented shoulder pain to their general practitioner, and followed them for 6 months. Our objective was to determine the shoulder pain related costs during the 6 months following first consultation for their complaints in general practice.

## Methods

### Recruitment

Between January 2001 and June 2003, 103 general practitioners (GPs) recruited patients at first consultation for a new episode of shoulder complaints in three geographic areas in the Netherlands (Amsterdam, Groningen and Maastricht).

In this study shoulder pain was defined according to the 1999 version of the Dutch guidelines for shoulder complaints, issued by the Dutch College of General Practitioners [14,15]. Shoulder pain was characterized as pain in the deltoid and upper arm region. Pain and stiffness were the prominent complaints. Pain and stiffness restricted the use of the arm and therefore limited daily activities, especially when using the hands above shoulder level. Lying on the affected shoulder was painful, indicating that severe shoulder pain could cause problems with sleeping.

Patients were selected if they were older than 18 years of age, and had not consulted their GP or received any form of treatment for the afflicted shoulder in the preceding three months. Sufficient knowledge of the Dutch language was required to complete written questionnaires. Exclusion criteria were severe physical or psychological conditions (i.e. fractures or luxation in the shoulder region; rheumatic disease; neoplasm; neurological or vascular disorders; dementia).

### Management of shoulder pain

The participating GPs were educated and trained to apply treatment according to the 1999 version of the Dutch guidelines for shoulder disorders issued by the Dutch College of General Practitioners[14,15]. The guidelines recommend giving information on the prognosis of shoulder pain, advice regarding provoking activities, and stepwise treatment consisting of paracetamol, Non-Steroidal Anti-Inflammatory Drugs (NSAIDs), corticosteroid injection or referral to physiotherapy. The participating GPs made the decision regarding the content of treatment based on duration and severity of pain and disability.

### Costs

Cost data were collected from a societal perspective, using a cost diary that has been shown to be valid and feasible for patient completion [16]. The diary was presented in a booklet form, containing instructions, an example of how to complete the diary, and a telephone number in case of questions. Patients were asked to complete five cost diaries during the entire follow-up period of 26 weeks, which was divided in five time periods: weeks 1–6, weeks 7–12, weeks 13–18, weeks 19–22, and weeks 23–26. Patients received a new diary by post at the beginning of a period and a return envelop for the previous diary. Patients were reminded by post after two weeks or telephoned after three weeks if they had not returned the previous diary. The cost diary included direct health care costs relevant to the treatment of shoulder complaints, such as visits to a general practitioner, physiotherapist, manual therapist, occupational therapist, 'Mensendieck' or 'Cesar' exercise therapist or complementary health therapists (e.g. acupuncturist), visits to a consultant in orthopedic surgery, neurology, rheumatology, or rehabilitation medicine, and hospitalization. Direct non-health care costs included out of pocket expenses, costs of performing extra activities (i.e. swimming, fitness, gymnastics), homecare and costs for paid and unpaid help. Indirect costs included costs of loss of production due to shoulder complaints, which was measured by sick leave from paid and unpaid work, and inability to perform usual activities and hobbies. Indirect costs for paid work were calculated using the friction cost method[17,18]. This method takes into account that sick workers can be replaced after a certain period of time, i.e. the friction period, depending on the elasticity of the labour market. In the Netherlands the friction period has been estimated at 123 days. Friction costs were based on the mean income of the Dutch population per gender and 5-year age group[17,18]. We used a shadow price for unpaid work of €8.60 per hour [17,18]. A complete overview of the unit costs we used is given in Table 1. Medication costs were based on the prices provided by the Royal Dutch Society for Pharmacy [19].

### Data analysis

We compared the baseline characteristics of patients who completed all cost diaries with those who did not and analysed possible differences using multiple logistic regression analysis. Health care consumption for shoulder pain and associated costs are presented in tables, for three time periods separately: short-term (1–6 weeks), intermediate term (7–12 weeks), and long-term follow-up (13–26 weeks). The arithmetic mean, standard deviations, maximum value (only for consumption) were computed for direct (health and non-health related care), and indirect costs. Despite the skewness in the distribution of costs, it is the arithmetic mean that is the most informative measure for health care policy makers [20,21]. Measures other than the arithmetic mean provide no information about the total cost of treating all patients. We also described the costs separately for patients with persistent symptoms after 6 months and for those reporting recovery. This 'Patient perceived recovery' was measured on an 8-point rating scale (very much detoriation, much detoriation, some detoriation, no change, some improvement, much improvement, very much improvement, fully recovered). Patients who did not report full recovery or very much improvement were denoted as having "persistent symptoms" The cut-off point was determined a priori, based on previous research by the project team [22,23].

The study was approved by the Medical Ethics Committee of the VU University Medical Center, Amsterdam, the Netherlands.

## Results

### Patients

A total of 587 patients were included in the cohort study. A total of 95 patients did not return one or more of their cost diaries. A multiple logistic regression analysis showed that patients with incomplete cost data were significantly (p < 0.10) younger (46 years versus 52 years) and had more concomitant neck pain (38% versus 35%). The results of this study were based on the 492 patients (84%) who completed all five cost diaries. Table 2 presents their baseline characteristics.

### Health care consumption and sick leave

Health care consumption and sick leave are presented in Table 3. The mean number of visits to GPs), specialists, allied health professionals, and complementary health therapists was low, as well as the mean consumption of non-health related care (home care, paid and unpaid help, extra activities). Productivity losses were not considered high as well, with a mean number of days sick leave from paid work of 2.8 days (sd ± 13) over a period of 6 months. Table 3 shows that most estimations have large standard deviations.

### Costs

The mean costs and standard deviations are presented in Table 4. During the 6 months after first consultation for shoulder pain, the mean total costs a patient generated were €689 (sd ± 1965). A large proportion (47%) of the total costs was due to indirect costs of productivity losses. Mean total costs due to sick leave from paid work in patients with a paid job were €523 (sd ± 2054). Treatment by a therapist (mostly physiotherapists, Table 3) accounted for 37% of the total direct costs. One patient underwent surgery for his shoulder complaints, and generated €2715, which reflects 1 day of hospitalization and the costs of a neck operation. The costs generated in the first six weeks (€276 ± 758) were almost equal to the costs generated in the final three months of the follow-up period (€257 ± 962). So, the highest costs per week were generated in the first 6 weeks after consultation. Table 5 shows that patients reporting persistent symptoms generated more than twice as much costs compared with patients reporting recovery after 6 months.

**Figure 1:**
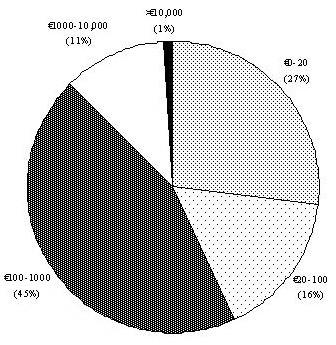
Distribution of the total costs (€) in the population (n = 492).

Figure 1 describes the distribution of the total costs in the population. A small proportion of the population (n = 61; 12%) generated more than €1000 per patient during 6 months after first consultation. This skewness was illustrated by the fact that median total direct costs were only €105 (Inter Quartile Range 19–317) and the median total indirect costs €0 (IQR 0–75). These 61 patients (12%) were responsible for 74% of the total costs. These small subset of the population differed considerably (>10%) at baseline from the total study population, reporting more sick leave at baseline in the preceding 2 months, more often strain due to usual activities as precipitating cause, more shoulder or neck complaints in the past, more concomitant neck, high back, or back pain, higher intensity of shoulder pain, more shoulder disability, and more shoulder and neck pain at physical examination (Table 2). The prevalence of persistent symptoms after 6 months in this subgroup was 70%. Sick leave from paid work in 6 months after consultation accounted for 61% of the total costs in this subgroup.

## Discussion

The present study is the first to evaluate the overall costs generated by patients presenting with shoulder pain in primary care. Healthcare consumption and sick leave did not seem to be alarmingly high in this primary care population with mean total costs of €689 per patient during the 6 months after first consultation. A small part (12%) of the population accounted for 74% of the total costs.

The response rate was high (84%) and differences at baseline between completers (all 5 five cost diaries) and non-completers (<5 cost diaries) were small. We expect that the differences in age and concomitant neck pain between completers and non-completers will not have substantially influenced our cost estimates.

There was a high percentage of patients who reported persistent symptoms after 6 weeks (70%) and 6 months (46%) in this population [24]. These rates are similar to those found in other studies carried out in primary care populations [10-12], which may strengthen the generalisability of our findings to other primary care patients with shoulder disorders. There was little health care consumption and shoulder pain related sick leave in this study. As a consequence shoulder pain related costs per patient were low. Only one patient reported 130 days of sick leave in the 6 months after consultation, which was more than the friction period of 123 days.

An explanation for the modest health care costs could be that general practitioners stick to the interventions recommended in the Dutch guidelines for shoulder disorders [14,15] (wait-and-see policy with pain medication, followed by injections), which are relatively inexpensive. Possibly, the fact that GPs were instructed to follow the guideline could have influenced the generalisability of our results because GPs who did not participate in our study subscribe other interventions, which could have resulted results in different costs estimates. The guideline, however, is not complicated and in line with daily practice (stepwise management regime). The guideline was issued in 1999, a few years before the inclusion of our study started. So, GPs who did not participate in the study could have been influenced by the guideline as much as GPs who participated in the study and followed a course during a half day. So, we do not expect this to strongly influence the generalisability of our findings.

The costs of physiotherapy represented a relatively large proportion of the direct health care costs, but accounted for only 14% of the total costs, as few patients were referred for therapy (Table 4). Indirect costs accounted for a large proportion (47%) of the total costs. Nevertheless, the total number of days sick leave per patients was small (2.8 days) over a period of 6 months. Possibly, factors such as shoulder pain, sleeping problems, or loss of function have caused loss of productivity in patients without sick leave from paid work. Our study does not provide information on the actual loss of productivity among those who kept on working regardless of the shoulder pain ("work presenteeism"), or costs related to hiring and training replacements, spill-over effects to co-workers, and compensatory mechanisms that may reduce or increase the overall productivity loss [25]. Clearly, a more adequate estimation of production loss is needed to improve the meaningfulness of future studies.

Similar to studies on low back pain [26], in this study a small proportion of the population (n = 61; 12%) caused a substantial part (74%) of the total costs. In this subgroup sick leave from paid work accounted for 61% of the total costs. In a prognostic study we found the following factors predicting sick leave from paid work in 6 months after consultation: sick leave in the preceding 2 months, shoulder pain, precipitating cause: strain due to usual activities and concomitant psychological complaints[27]. Table 2 shows that the subgroup who generated most costs, not surprisingly, also showed higher scores on these variables compared to the total population (Table 2). Table 2 also shows substantial differences between the groups regarding shoulder or neck pain at physical examination, and concomitant back pain at baseline. This seems plausible as these factors were shown to be of predictive value for persistent shoulder pain after 6 weeks and 6 months in our cohort [24].

In this study we were able to include a follow-up of 26 weeks. It is possible that prolonged and recurrent pain episodes generate additional costs for more expensive care, e.g. diagnostic imaging and surgical treatment, including hospitalization. Given the poor prognosis of shoulder pain (approximately 40% of patients report persistent symptoms after 12 to 18 months [10-12]. higher health care costs and productivity losses may be expected when follow-up times are longer. In the 6 months following first consultation, few costs were made due to referrals to other health care providers, additional diagnostic procedures, or surgery. We expect these kinds of health care expenses to occur in the long-term in a small subset of the population. In our inception cohort patients with fractures, dislocation, or previous surgery were not included. These patients are not included in this study, but may generate substantial costs in current practice.

Information on the costs associated with health care use and loss of productivity in patients with shoulder pain is very scarce, and therefore a comparison with other studies is difficult. In the framework of this cohort study a randomised controlled trial on the effectiveness of manipulative therapy has been carried out [23]. This trial was similar regarding outcome assessments and length of follow-up. The control group of this trial (n = 71), who also received usual care according to the Dutch general practice guidelines, generated slightly lower costs (mean total costs €555) compared to our cohort.

In conclusion, the total costs in the 26 weeks after first consultation for shoulder pain, mostly generated by a small part of the population, are not alarming. However, after 26 weeks 46% of the patients still reported persistent symptoms. More extensive research with a longer follow-up to monitor these patients is needed, as they possibly generate substantial costs. Given the high incidence of shoulder pain (12/1000/year) [7-9] in general practice the total costs to society could be higher than presented in this study. It may be important to also include patients with fractures, dislocation, or previous surgery to estimate the total costs of illness for patients consulting with shoulder pain in primary care.

## Competing interests

The author(s) declare that they have no competing interests.

## Authors' contributions

TK carried out the analysis and drafted the manuscript. MWT participated in the design and drafting of the study. GJH participated in the design and drafting of the study. LMB participated in the design and drafting of the study. DAW participated in the design and drafting of the study. All authors read and approved the final manuscript.

## Pre-publication history

The pre-publication history for this paper can be accessed here:



## Figures and Tables

**Table 1:** Cost used in the economic evaluation

**Costs**	**€**
*Direct health care costs (per visit)*	
General practitioner (max 10 min)	18.97
Manual therapist (max 20 min)	19.65
Other therapists (max 30 min) (physiotherapist, 'Mensendieck', 'Cesar' and occupational therapist)	19.65
Specialists (orthopaedist, neurologist, rheumatologist, and physician for rehabilitation medicine)	44.21
Hospitalisation (per day)	337.00
	
*Direct non-health care costs (per hour)*	
Alternative therapists (acupuncturist, homeopath, chiropractor, and others)	As indicated by patient
Home care	8.60
Paid help at home	8.60
Help from partner/relatives/friends	8.60
Extra activities (i.e. swimming, fitness, gymnastics)	8.60
	
*Indirect costs (per hour)*	
Sick leave from paid work	FCM
Sick leave from unpaid work	8.60
Unable to perform usual activities	8.60
Unable to perform hobbies	8.60

€: Euro's (€1 = 1.30 US Dollars, 02/02/2005); FCM: friction cost method

**Table 2:** Baseline characteristics of patients with shoulder pain

**Variable**	**n = 492**	n = 61*
*Demographic*		
Age (years); mean (SD)	52 (14)	50 (14)
Gender: male; n (%)	245 (50)	25 (41)
Paid work; n (%)	350 (60)	38 (62)
		
*Disease characteristics*		
Duration of current shoulder complaints; n (%)		
0–6 weeks	179 (37)	18 (30)
7–12 weeks	117 (24)	15 (25)
>12 weeks	195 (40)	28 (46)
Sick leave at baseline in preceding 2 months^$^; n (%)		
0 weeks	254 (74)	21 (57)
≤1 weeks	44 (13)	5 (13)
>1 weeks	46 (13)	11 (30)
Gradual onset; n (%)	310 (63)	36 (59)
Precipitating cause; n (%)		
Strain/overuse: usual activities; n (%)	115 (23)	22 (36)
Shoulder complaints in the past; n (%)	291 (59)	42 (69)
Neck complaints in the past; n (%)	251 (51)	41 (68)
Dominant side involved; n (%)	302 (61)	42 (69)
Comorbid psychological complaints; n (%)	42 (9)	9 (15)
Concomitant musculoskeletal complaints; n (%)		
Neck/high back	173 (35)	35 (57)
Low back pain	117 (24)	24 (39)
Upper extremity	152 (31)	21 (34)
Shoulder pain (0–10); mean (SD)	4.7 (2.3)	6.2 (1.9)
Shoulder disability (0–100); mean (SD)	60.0 (23.8)	72.0 (20.2)
		
*Physical examination*		
Pain shoulder with movement (0–18); median (IQR)	6 (4–6)	8 (6–8)
Pain neck with movement (0–18); median (IQR)	0 (0-0)	3 (0–2)
		
*Physical factors*		
Dynamic physical workload (0–5); median (IQR)	1 (0–1)	1 (1-1)
Repetitive movements; n (%)	316 (64)	43 (71)

*Subgroup of patients generating costs of >€1000 in 6 months. SD = standard deviation; IQR = Inter quartile range

^$ ^Sick leave due to shoulder pain

**Table 3:** Consumption of healthcare resources and sick leave from work during 26 weeks after first consultation for shoulder complaints (n = 492)

	**week 1–6**	**week 7–12**	**week 13–26**	**Total (week 1–26)**
**Direct health care**				
General practitioner [no. of visits]	1.6 ± 0.9 (6)	0.2 ± 0.6 (4)	0.3 ± 0.7 (5)	2.1 ± 1.5 (9)
Allied health professionals [no. of visits]				
Physiotherapist	1.5 ± 3.0 (13)	1.1 ± 2.6 (16)	1.5 ± 4.1 (28)	4.1 ± 7.6 (45)
Manual therapist	0.2 ± 1.0 (11)	0.2 ± 0.9 (9)	0.2 ± 1.1 (11)	0.5 ± 2.2 (20)
Other therapists	0.1 ± 0.5 (6)	0.0 ± 0.3 (4)	0.1 ± 0.7 (12)	0.2 ± 1.2 (19)
Specialists [no. of visits]	0.0 ± 0.3 (3)	0.0 ± 0.2 (2)	0.1 ± 0.5 (5)	0.2 ± 0.8 (7)
Hospitalisation [days]	-	-	0.0 ± 0.0 (1)	0.0 ± 0.0 (1)
				
**Direct non-health care**				
Alternative therapists [no. of visits]	0.1 ± 0.5 (6)	0.1 ± 0.5 (8)	0.2 ± 0.9 (11)	0.3 ± 1.6 (17)
Home care [hours]	0.1 ± 1.2 (22)	0.0 ± 0.8 (15)	0.2 ± 2.0 (32)	0.3 ± 3.1 (51)
Paid help [hours]	0.4 ± 3.6 (48)	0.5 ± 4.3 (60)	0.7 ± 5.7 (80)	1.7 ± 12.4 (176)
Help from partner/relatives/friends [hours]	1.1 ± 8.7 (152)	0.9 ± 8.9 (168)	1.0 ± 6.3 (72)	3.2 ± 16.1 (212)
Extra activities [hours]	0.8 ± 3.9 (44)	1.0 ± 5.3 (93)	1.4 ± 6.9 (95)	3.2 ± 11.6 (121)
				
**Indirect**				
Sick leave from paid work [days]	1.1 ± 5.0 (42)	0.6 ± 3.6 (30)	1.1 ± 6.3 (63)	2.8 ± 13.0 (123)
Sick leave from unpaid work [days]	1.9 ± 9.6 (120)	1.4 ± 9.9 (168)	1.6 ± 9.5 (104)	4.9 ± 24.2 (322)
Unable to perform usual activities [hours]	1.4 ± 7.7 (106)	0.9 ± 6.7 (108)	1.0 ± 7.3 (104)	3.2 ± 16.1 (212)
Unable to perform hobbies [hours]	2.8 ± 9.9 (120)	1.1 ± 5.7 (84)	1.3 ± 6.8 (75)	5.3 ± 16.3 (139)

Presented are group means, standard deviations and the maximum value between brackets.

Other therapists: Mensendieck, Cesar, occupational therapists, or other.

Specialists: orthopaedist; neurologist; rheumatologist; physician for rehabilitation medicine, or other.

Alternative therapists: acupuncturist; homeopath; chiropractor; hypnotist; magnetism therapist, or other.

**Table 4:** Mean costs (€) made in 26 weeks after first consultation for shoulder complaints (n = 492)

	**week 1–6**	**week 7–12**	**week 13–26**	**Total (week 1–26)**
**Direct health care costs**	**69 ± 66**	**32 ± 57**	**52 ± 179**	**152 ± 232**
General Practitioner	31 ± 17	4 ± 54	5 ± 14	40 ± 29
Allied health professionals	34 ± 62	25 ± 53	34 ± 87	93 ± 158
Specialists	2 ± 12	1 ± 9	5 ± 26	8 ± 36
Prescribed medication	2 ± 6	1 ± 3	1 ± 4	4 ± 10
Hospitalisation	-	-	6 ± 138	6 ± 138
				
**Direct non-health care costs**	**30 ± 102**	**26 ± 108**	**41 ± 146**	**98 ± 293**
Alternative therapists	5 ± 33	3 ± 28	8 ± 55	16 ± 94
Over-the-counter medication	2 ± 9	1 ± 9	4 ± 51	7 ± 58
Out-of-pocket expenses	2 ± 16	1 ± 8	1 ± 6	4 ± 21
Home care	1 ± 10	0.4 ± 7	2 ± 17	3 ± 27
Paid help	4 ± 31	5 ± 37	6 ± 49	15 ± 107
Help from partner/relatives/friends	10 ± 75	7 ± 76	8 ± 54	25 ± 168
Extra activities	7 ± 34	8 ± 45	12 ± 59	28 ± 100
				
**Total direct costs**	**99 ± 130**	**58 ± 134**	**93 ± 244**	**250 ± 408**
				
**Indirect costs**				
Sick leave from paid work	125 ± 654	69 ± 461	130 ± 790	324 ± 1635
Sick leave from unpaid work	16 ± 83	12 ± 85	13 ± 82	42 ± 208
Unable to perform usual activities	12 ± 66	7 ± 57	9 ± 63	28 ± 139
Unable to perform hobbies	24 ± 85	10 ± 49	12 ± 59	46 ± 140
				
**Total indirect costs**	**177 ± 711**	**99 ± 505**	**164 ± 829**	**439 ± 1751**
				
**Total costs**	**276 ± 758**	**156 ± 564**	**257 ± 962**	**689 ± 1965**

Presented are group means, and standard deviations.

**Table 5:** Mean costs (€) and standard deviations in patients with persistent symptoms versus those in recovered patients after 6 and 26 weeks (n = 492)

	**week 1–6**		**week 1–26**	
	*Persistent symptoms*	*Recovered*	*Persistent symptoms*	*Recovered*
				
Direct health care costs	82 ± 71	48 ± 49	206 ± 301	107 ± 133
Direct non-health care costs	37 ± 115	20 ± 82	133 ± 316	70 ± 274
Total direct costs	120 ± 144	68 ± 98	339 ± 477	177 ± 325
Indirect costs	208 ± 727	127 ± 679	628 ± 2049	279 ± 1458
				
Total costs	328 ± 780	195 ± 696	966 ± 2334	457 ± 1573

Presented are group means, and standard deviations.
